# The range of the required anterolateral cortex osteotomy distance varied widely in biplanar open wedge high tibial osteotomy

**DOI:** 10.1186/s12891-022-05283-z

**Published:** 2022-04-06

**Authors:** Shuntaro Nejima, Ken Kumagai, Shunsuke Yamada, Masaichi Sotozawa, Shuhei Natori, Kei Itokawa, Yutaka Inaba

**Affiliations:** Department of Orthopaedic Surgery, Yokohama City University School of Medicine, 3-9 Fukuura, Kanazawa-ku, Yokohama, 236-0004 Japan

**Keywords:** Open wedge high tibial osteotomy, Transverse cut, Ascending cut, Lateral hinge, Anterolateral cortex

## Abstract

**Background:**

To evaluate the anterolateral cortex distance between the lateral edge of the flange and hinge point in surgical simulations of biplanar open wedge high tibial osteotomy (OWHTO) using computed tomography (CT) images.

**Methods:**

A total of 110 knees treated with OWHTO for medial knee osteoarthritis with varus malalignment were enrolled. Surgical simulations of biplanar OWHTO, including the transverse and ascending cuts, were performed in the standard manner using preoperative CT images. The distance between the lateral edge of the flange and the hinge point was measured. In addition, another plane of the ascending cut was defined through the hinge point. The angle between these two planes of the ascending cut was measured in the axial plane.

**Results:**

The mean anterolateral cortex distance was 9.4 ± 4.6 mm (range, − 1.5 mm – 20.3 mm). In 3 knees, osteotomy of the anterolateral cortex was not needed. The mean value of the angle between the two ascending cut planes was 8.4 ± 3.6° (range, − 2.1° – 14.8°), which meant that osteotomy of anterolateral cortex was not needed when the ascending cut was performed at this angle. Moreover, these two values increased when the flange thickness was changed from one-third to one-fourth of the anteroposterior tibial diameter or the angle between the transverse and ascending cuts was changed from 110° to 120°.

**Conclusions:**

In biplanar OWHTO, anterolateral cortex osteotomy would be required. However, the range of the required anterolateral cortex osteotomy distance varied widely and the required anterolateral cortex osteotomy distance depended on the flange thickness and the angle between the transverse and ascending cuts. In addition, change of the ascending cut plane can change the necessity of anterolateral cortex osteotomy.

## Background

**Fig. 1:**
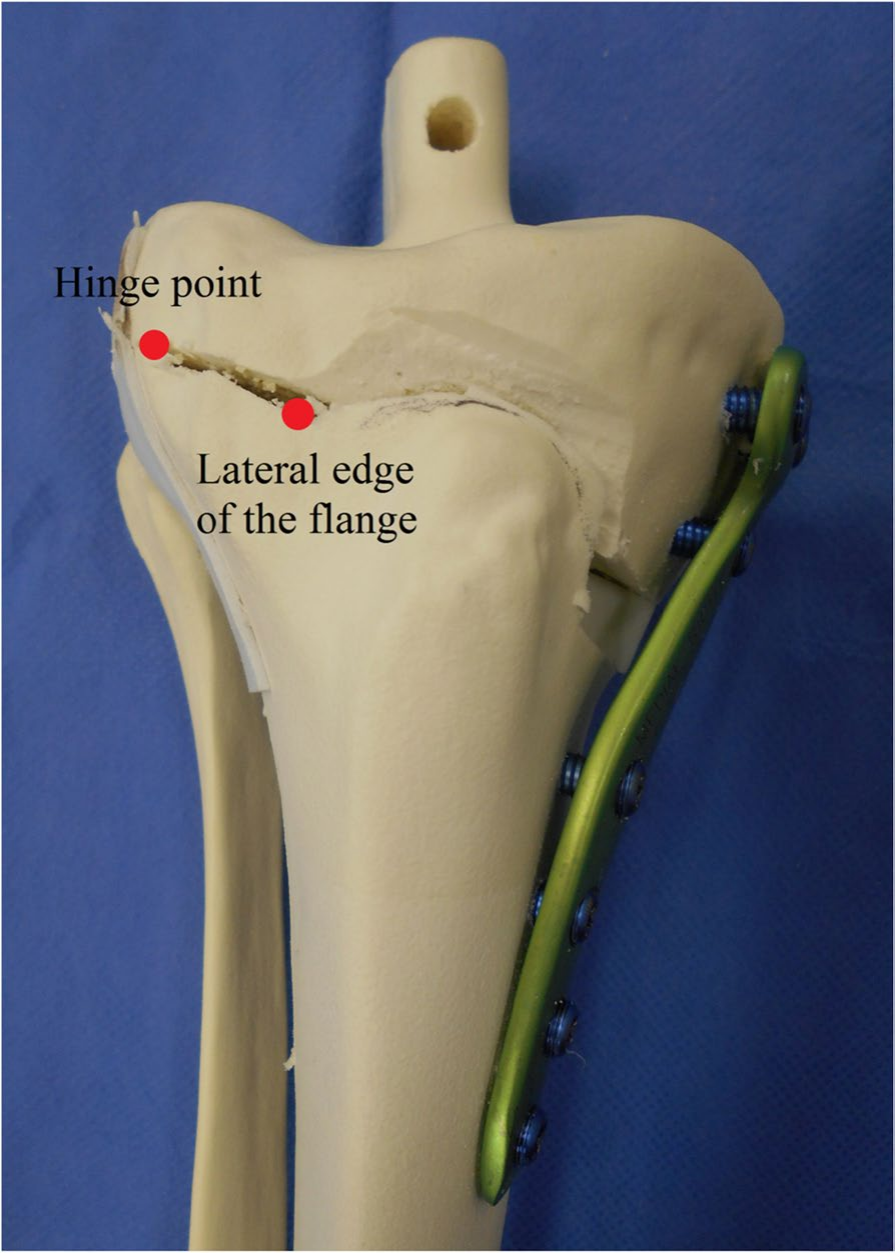
The anterolateral cortex between the lateral edge of the flange and the hinge point should be cut to open the osteotomy site in biplanar open wedge high tibial osteotomy

Open wedge high tibial osteotomy (OWHTO) is a well-established procedure for medial knee osteoarthritis (OA) with varus malalignment [1–3]. However, various complications, including lateral hinge fracture, are caused by inappropriate surgical techniques [4–6]. An abnormal position of the lateral hinge may lead to lateral hinge fracture [7, 8]. In addition, the transverse cut needs to be in the appropriate direction to prevent vascular injury [9, 10]. Thus, accurate and careful osteotomy is needed to prevent these complications. In a typical case of biplanar OWHTO, the anterolateral cortex between the lateral edge of the flange and the hinge point should be cut to open the osteotomy site (Fig. 1). Nonetheless, in some cases, the osteotomy site can be opened without this procedure. In these cases, additional osteotomy of the anterolateral cortex may lead to potential injury to normal structures such as the lateral hinge. However, the shape of this area has not been evaluated. Thus, this study focused on the anterolateral cortex between the lateral edge of the flange and the hinge point.

The purpose of this study was to evaluate the anterolateral cortex distance between the lateral edge of the flange and hinge point in surgical simulations of biplanar OWHTO using computed tomography (CT) images. It was hypothesized that the required anterolateral cortex osteotomy distance in biplanar OWHTO varies widely and that osteotomy of this area is not always needed.

## Methods

A total of 97 patients (126 knees) who underwent OWHTO for medial knee OA with varus malalignment from July 2011 to January 2018 were enrolled. CT images were obtained preoperatively. Patients with OA of the hip (2 knees), patients with a history of surgical treatment of the lower limbs (12 knees) and patients who underwent concomitant procedures such as anterior cruciate ligament reconstruction and tibial tubercle transfer with OWHTO (2 knees) were excluded. Thus, 87 patients (110 knees) met the inclusion criteria for this study. The demographic data are shown in Table 1.

**Table 1:** Patients’ demographic characteristics

Knees, n	110
Age, y	65.7 ± 7.7 (45 – 80)
Height, cm	157.2 ± 7.5 (138.5 – 182.9)
Weight, kg	63.5 ± 10.3 (43.8 – 92.4)
Body mass index, kg/m^2^	25.7 ± 3.7 (19.0 – 40.2)
Side, left/right	51/59
Sex, female/male	81/29
Ahlbӓck grade 1/2/3	81/21/8

Data are presented as the mean ± standard deviation with the range in parentheses

### Simulation of OWHTO on CT images

**Fig. 2:**
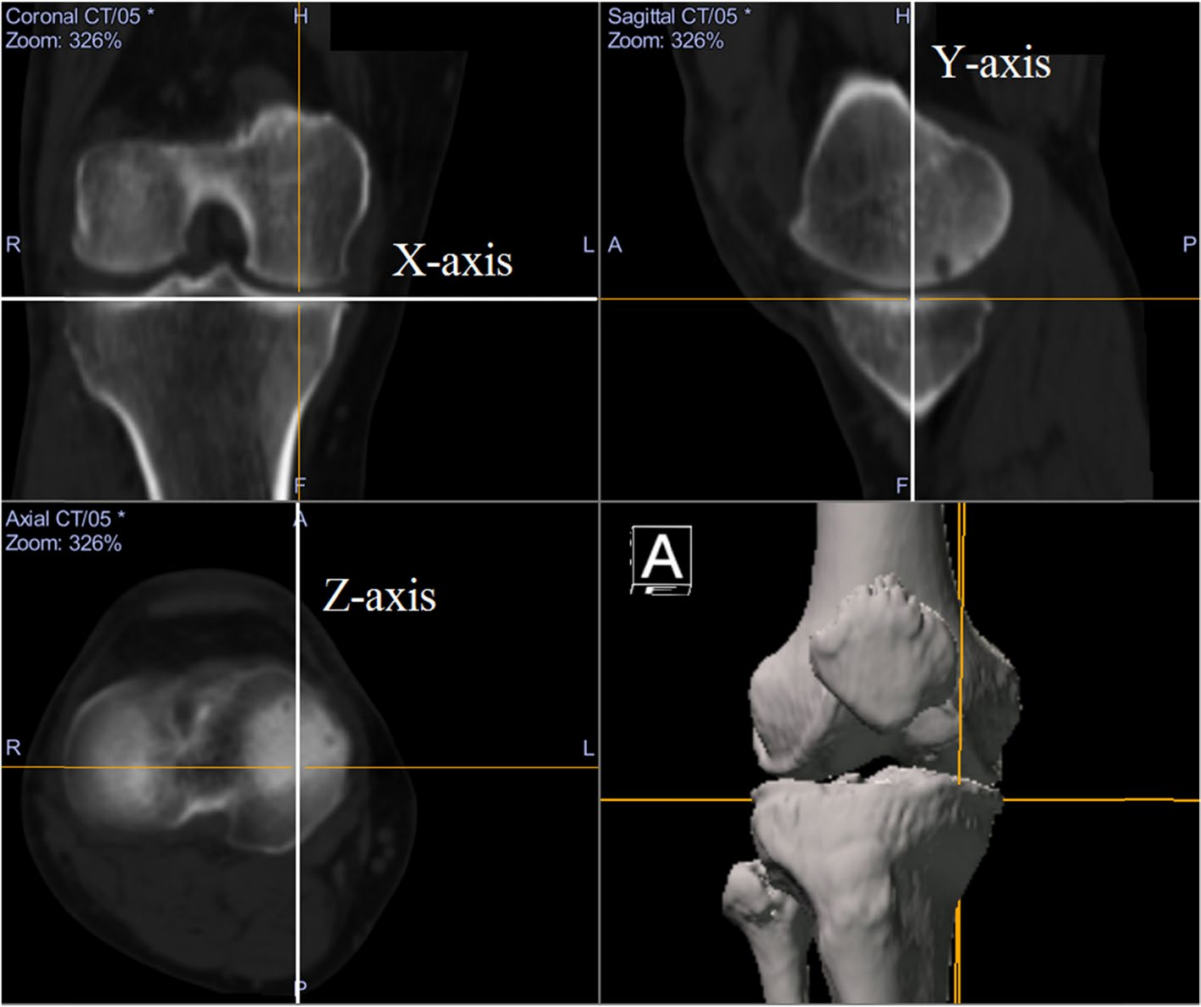
The X-axis was defined as the line parallel to the tibial plateau in the coronal plane. The Y-axis was defined as the line perpendicular to the medial tibial plateau in the sagittal plane. The Z-axis was defined as the anteroposterior axis of the knee in which the patella was located in the centre of the distal femur

**Fig. 3:**
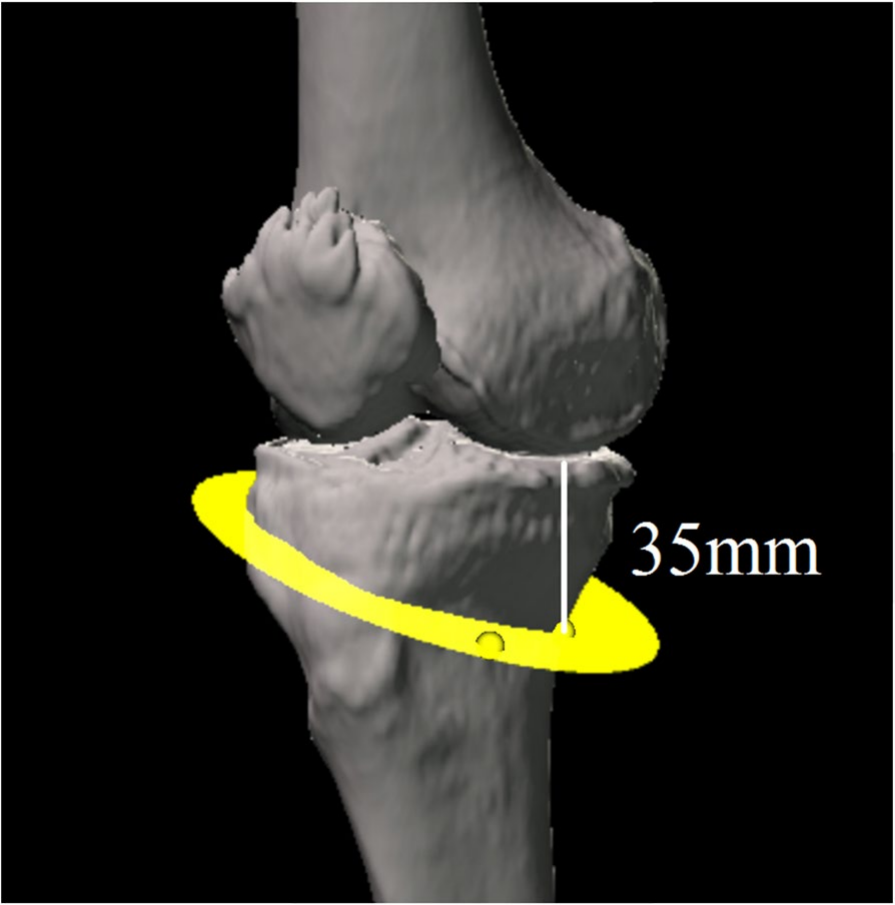
The plane of the transverse cut was made 35 mm distal to the medial tibial plateau and extended to the tip of the fibular head

**Fig. 4:**
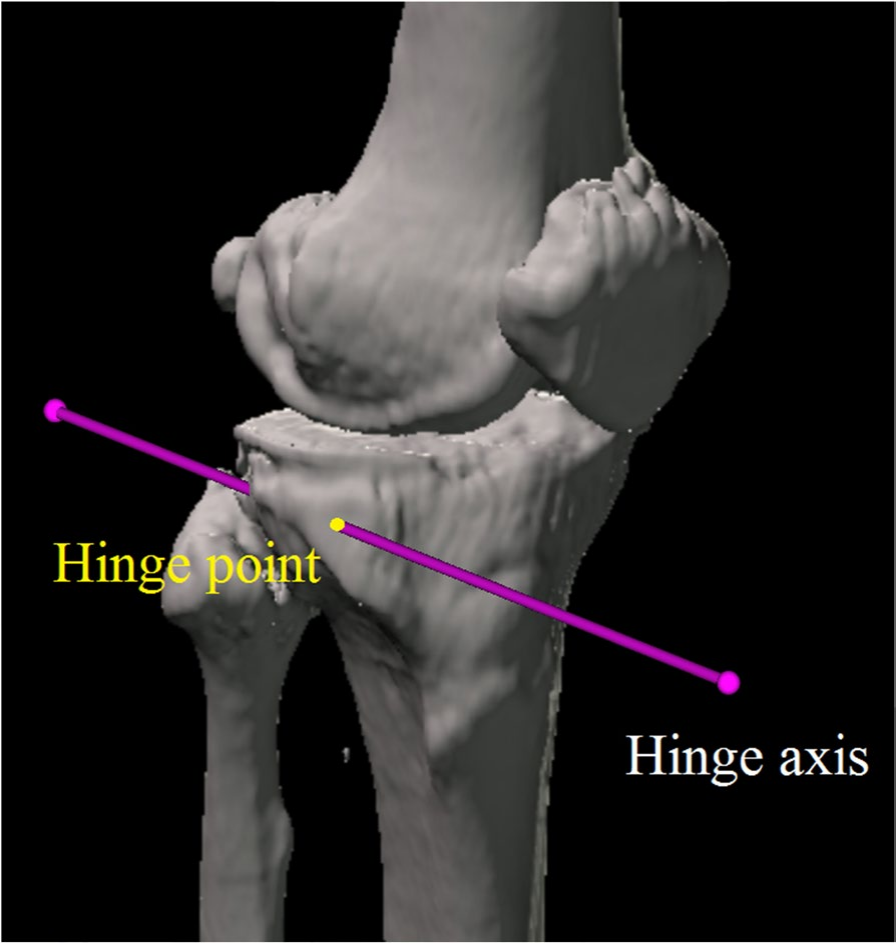
The hinge axis was defined as the line parallel to the Z-axis and 5 mm medial to the lateral tibial cortex on the plane of the transverse cut. The hinge point was defined as the intersection point of the hinge axis and the anterior surface of the tibia

**Fig. 5:**
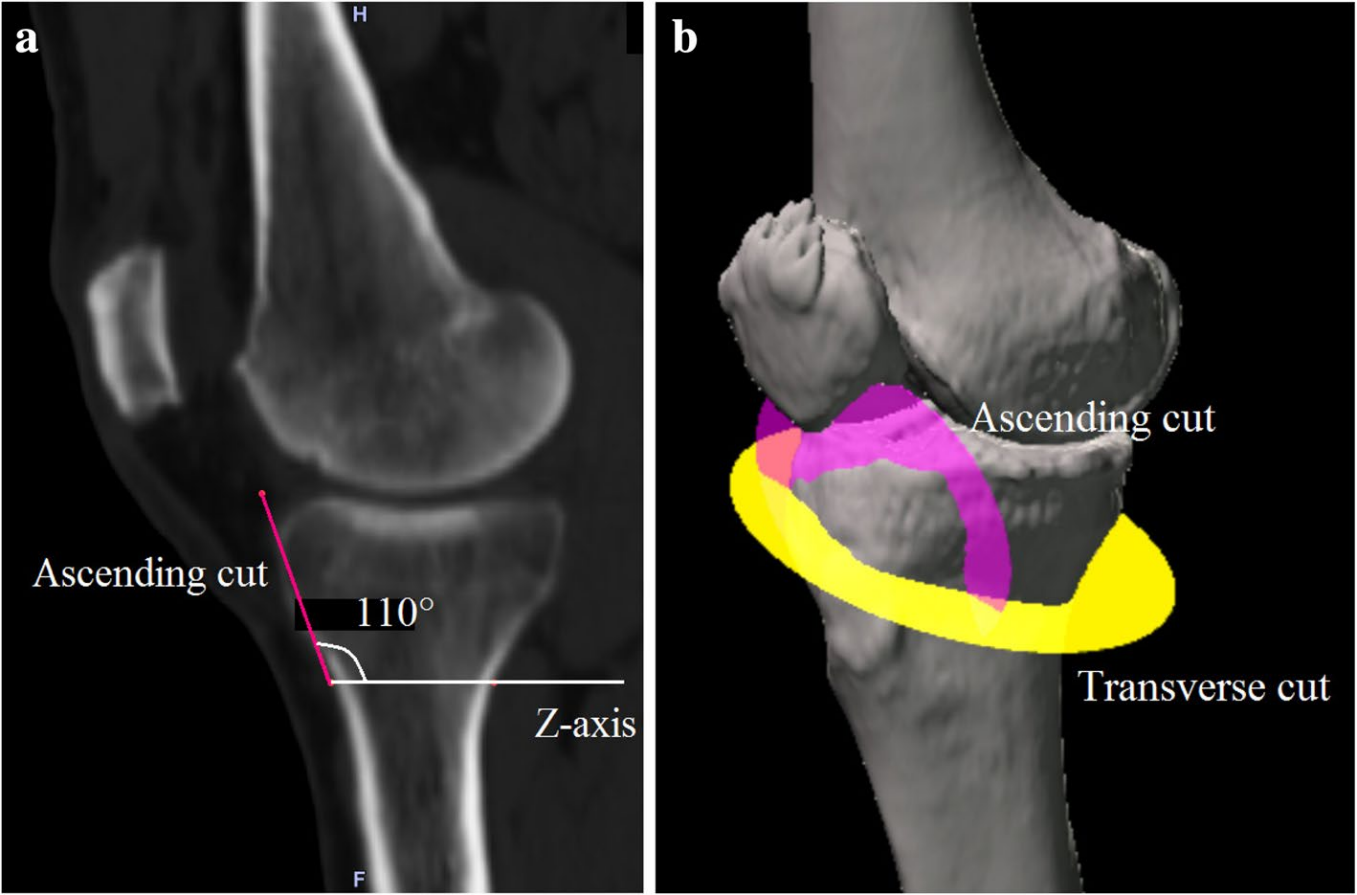
The ascending cut was defined as the plane angled 110° to the XZ plane (a), and one-third of the anteroposterior tibial diameter at 35 mm below the medial tibial plateau was left as the flange (b)

Long-leg CT images (1.5-mm-thick slices) were obtained with the patients lying supine on a SOMATOM Sensation 16 scanner (Siemens, Munich, Germany) for preoperative deformity analysis. The data were imported into Orthomap 3D (Stryker, Kalamazoo, MI), which enabled the selection of anatomical landmarks and the measurement of three-dimensional linear and angular parameters by simultaneously showing the sagittal, coronal, and axial planes [11]. The X-axis was defined as the line parallel to the tibial plateau in the coronal plane. The Y-axis was defined as the line perpendicular to the medial tibial plateau in the sagittal plane. The Z-axis was defined as the anteroposterior axis of the knee in which the patella was located in the centre of the distal femur (Fig. 2). The plane of the transverse cut was made 35 mm distal to the medial tibial plateau and extended to the tip of the fibular head [2, 12] (Fig. 3). The hinge axis was defined as the line parallel to the Z-axis and 5 mm medial to the lateral tibial cortex in the plane of the transverse cut [13]. The hinge axis was parallel to Z-axis to maintain posterior tibial slope after surgery [14]. The hinge point was defined as the intersection point of the hinge axis and the anterior surface of the tibia (Fig. 4). The ascending cut was defined as the plane angled 110° to the XZ plane [15] (Fig. 5a), and one-third of the anteroposterior tibial diameter 35 mm below the medial tibial plateau was left as the flange [1] (Fig. 5b).

### Measurements of the required anterolateral cortex osteotomy distance

**Fig. 6:**
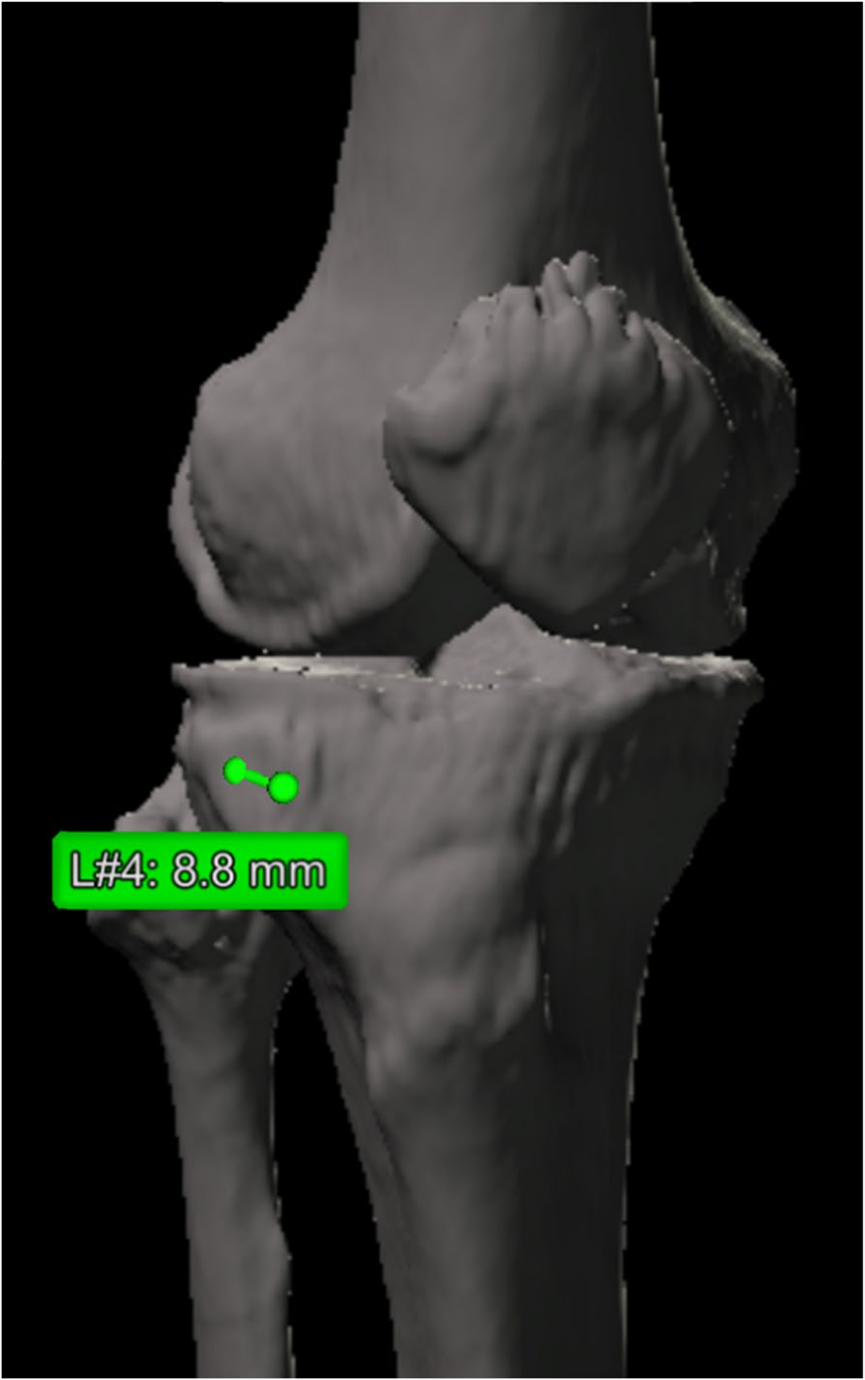
The distance between the lateral edge of the flange and the hinge point was measured

The lateral edge of the flange was defined as the lateral intersection between the planes of the transverse and ascending cuts on the anterior tibial surface. The distance between the lateral edge of the flange and the hinge point was measured (Fig. 6). A distance less than or equal to 0 means that osteotomy of the anterolateral cortex was not needed.

### Definition of the plane of the ascending cut through the hinge point

**Fig. 7:**
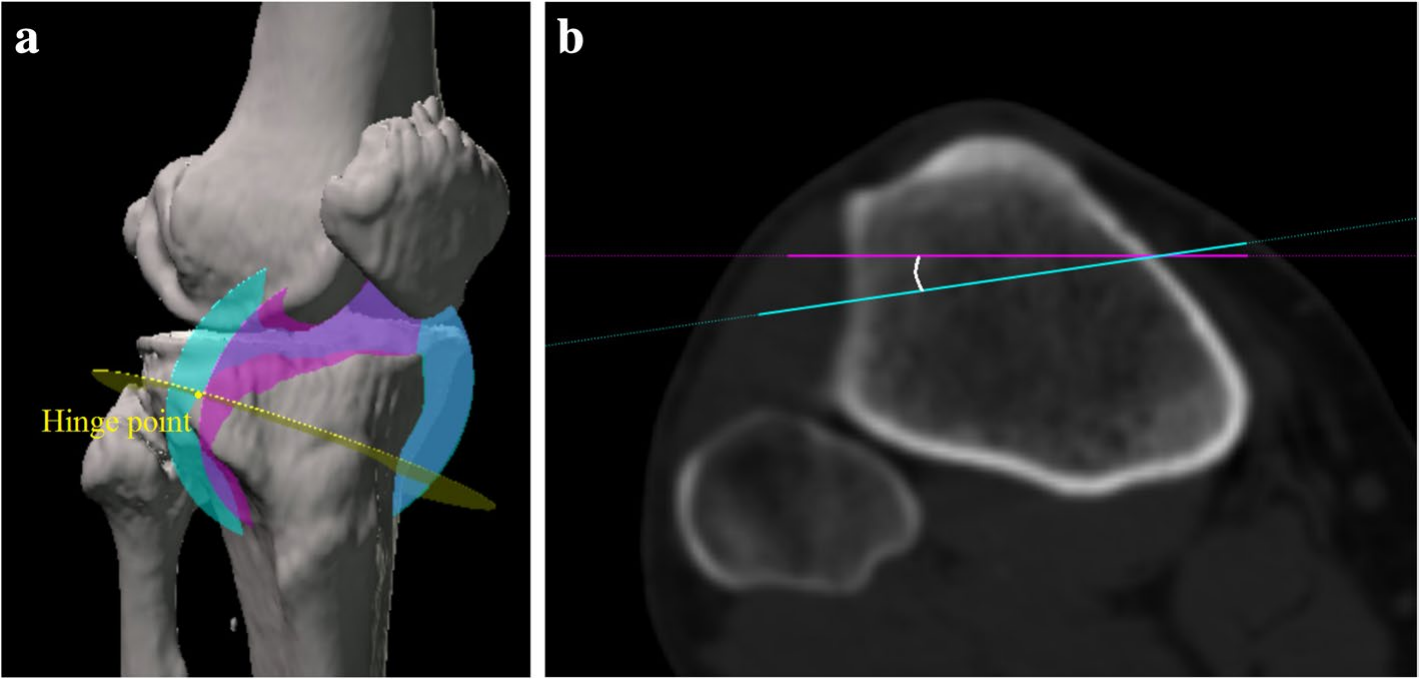
Another plane of the ascending cut was defined through the hinge point. The starting position of this new ascending cut was the same as the original one, and this plane was made to include the hinge point (a). Then, the angle between the plane of this transverse cut and the original one was measured in the XZ plane (b)

Another plane of the ascending cut was defined through the hinge point. The starting position of this new ascending cut was the same as that mentioned above, and this plane was made to include the hinge point (Fig. 7a). Then, the angle between this ascending cut and the original cut was measured in the XZ plane (Fig. 7b). Osteotomy of the anterolateral cortex was not needed when the ascending cut was performed at this angle.

### Measurements in different settings

In addition to the above setting, the required anterolateral cortex osteotomy distance and the angle between the two ascending cut planes were measured when the flange thickness was one-fourth of the anteroposterior tibial diameter and the angle between the transverse and ascending cuts was 110°, and when the flange thickness was one-third of the anteroposterior tibial diameter and the angle between the transverse and ascending cuts was 120°, respectively. This retrospective study was approved by the institutional review board of Yokohama City University (B210200009), and written informed consent was obtained from each patient.

### Statistical analysis

The data are expressed as the means ± standard deviations, and the Shapiro–Wilk test was used to confirm the normality of the data. To evaluate the effect of the flange thickness and the angle between the transverse and ascending cuts on the measured values, the required anterolateral cortex osteotomy distance and the angle between the two ascending cut planes in the basic setting were compared to those in the two additional settings using paired t-test. All statistical analyses were performed using IBM SPSS for Windows, version 21.0 (IBM Corporation, Armonk, NY, USA). A power analysis was performed on paired t-test (effect size = 0.3, significance level = 0.05, sample size = 110) using G*Power version 3.1.9.2 (Heinrich-Heine-Universität, Düsseldorf, Germany). A post hoc power analysis resulted in a power of 0.88. To assess the reproducibility of the anterolateral cortex distance between the lateral edge of the flange and hinge point and the angle between the two ascending cut planes mentioned above, 20 knees were randomly selected, and intra- and interobserver reliability were assessed by determining the intraclass correlation coefficients (ICCs) for each measurement.

## Results

The mean anterolateral cortex distance was 9.4 ± 4.6 mm (range, − 1.5 mm – 20.3 mm). In 3 knees (2.7%), this distance was less than or equal to 0, which meant that osteotomy of the anterolateral cortex was not needed. The mean value of the angle between the two ascending cut planes was 8.4 ± 3.6° (range, − 2.1° – 14.8°). These two values increased significantly when the flange thickness was changed from one-third to one-fourth of the anteroposterior tibial diameter or the angle between the transverse and ascending cuts was changed from 110° to 120° (Table 2). The intra- and interobserver ICC values were 0.87 and 0.79 for the distance between the lateral edge of the flange and hinge point and 0.88 and 0.84 for the angle between two planes, respectively. The width of the 95% confidence interval for the anterolateral cortex distance was 1.8 mm according to the sample size and standard deviation in the present study.

**Table 2:** CT data

	Basic setting	Flange thickness is one-fourth of the anteroposterior tibial diameter	Angle between the transverse and ascending cuts is 120°
Required anterolateral cortex osteotomy distance, mm	9.4 ± 4.6 (− 1.5 – 20.3)	14.9 ± 5.1 (3.2 – 24.4)^a^	15.2 ± 5.1 (0.8 – 24.4)^a^
Angle between the two ascending cut planes, °	8.4 ± 3.6 (− 2.1 – 14.8)	12.2 ± 3.6 (2.3 – 21.5)^a^	13.0 ± 3.9 (0.8 – 22.9)^a^

Data are presented as the mean ± standard deviation with the range in parentheses

^a^Significant difference compared with basic setting, *P* < 0.001

## Discussion

The most important finding of this simulation study was that the anterolateral cortex osteotomy was needed in 97.3% and not needed in only 2.7% in biplanar OWHTO. Meanwhile, the range of the required anterolateral cortex osteotomy distance varied widely. In addition, osteotomy was not needed if the ascending cut tilted against the coronal plane. Moreover, the required anterolateral cortex osteotomy distance depended on the flange thickness and the angle between the transverse and ascending cuts. In previous studies, appropriate methods of osteotomy for preventing complications have been reported [7–10]. However, osteotomy of the anterolateral cortex has not been evaluated. This study revealed that the anterolateral cortex osteotomy was needed in most knees and the range of the required osteotomy distance varied widely in biplanar OWHTO.

In biplanar OWHTO, theoretically, the anterolateral cortex between the lateral edge of the flange and the hinge point should be cut to open the osteotomy site. However, in clinical practice, the osteotomy site can sometimes be opened without this procedure. The present study revealed that osteotomy of the anterolateral cortex was not needed in 3 knees (2.7%), even for osteotomy performed in a standard manner. Whether common anatomical features exist among these knees is unclear. Theoretically, this procedure is not needed if the hinge point is positioned in front of the ascending cut plane. The gentle slope of the anterolateral cortex in the proximal tibia may make anterolateral cortex osteotomy unnecessary. In addition, the results of the present study revealed that osteotomy of this area was not needed if the ascending cut was angled to the coronal plane (mean angle was 8.4°). The ascending cut might tend to tilt against the coronal plane in real surgery. The angle between the ascending cut and posterior aspect of the tibia was approximately 30° in a previous study [16]. Surgeons should recognize the possibility of an increasing number of cases in which osteotomy of the anterolateral cortex is not needed in real surgical cases.

The results of the present study revealed that the required anterolateral cortex osteotomy distance and the angle between two ascending cut planes increased when the flange thickness was changed from one-third to one-fourth of the anteroposterior tibial diameter or the angle between the transverse and ascending cuts was changed from 110° to 120°. In these settings, the anterolateral cortex osteotomy was needed in all knees because the minimum value of the required anterolateral cortex osteotomy distance was greater than 0. Although the settings adopted in the present study were based on previous studies and these settings were thought to be reasonable, the thickness and direction of the ascending cut can be more personalized depending on the shape of the proximal tibia in real surgery. Surgeons should recognize that the range can be wider in real surgery than the surgical simulation in the present study.

Excessive osteotomy of the anterolateral cortex leads to potential injuries to normal structures such as the lateral hinge. Moreover, insufficient osteotomy of this area may lead to difficulty in opening the osteotomy site. In addition, if the anterolateral cortex is not cut, the hinge position will tilt anterolaterally in the axial plane. Consequently, the posterior tibial slope will decrease after opening the osteotomy site [17]. Excessive decrease of posterior tibial slope leads to overload on the posterior cruciate ligament [18]. Thus, accurate and careful osteotomy of this site is needed. However, it is difficult to cut the anterolateral cortex accurately because the range of the required anterolateral cortex osteotomy distance varied widely and because this area cannot be seen directly during surgery. Blind osteotomy of this area using a chisel or sawblade should be avoided. Tilting the ascending cut to the coronal plane or increasing the thickness of the flange intentionally could save the necessity for osteotomy of this area. However, normal structures such as the lateral hinge or tibial plateau could be damaged by these procedures. In addition, these procedures could lead to retro-tubercular gap widening and lateral hinge fracture [16]. Thus, tilting the ascending cut to the coronal plane or increasing the thickness of the flange intentionally should be avoided.

Recently, the utility of computer navigation or patient specific instrumentation (PSI) in OWHTO was reported. OWHTO with computer navigation reduced outliers of alignment correction in the coronal plane compared to that with conventional technique [19]. Although there was no significant difference of accuracy of alignment correction between PSI and conventional technique [20], OWHTO with PSI reduced the surgical and fluoroscopic times compared to that with conventional technique [21]. In addition, there was no learning curve for achieving the planned correction in OWHTO with PSI [22]. In general, OWHTO with these techniques was planned using CT images. Although the planning of the anterolateral cortex osteotomy does not seem to be taken into consideration in these techniques, the necessity of anterolateral cortex osteotomy could be expected with surgical simulation using preoperative CT images. The CT planning of OWHTO with computer navigation or PSI would lead to more accurate osteotomy including anterolateral cortex and consequent more favorable clinical outcome.

There was a limitation in this study. Because this study is based on surgical simulations using CT images, whether excessive or insufficient osteotomy of the anterolateral cortex leads to complications such as lateral hinge fracture and worse clinical outcome was unclear. However, the distance between the lateral edge of the flange and hinge point has not been evaluated in previous studies, and it is clear that accurate osteotomy is important in OWHTO. Whether accurate osteotomy of this area, as mentioned above, leads to a decrease in complications or worse clinical outcomes should be elucidated in the future.

In daily clinical practice, surgeons should recognize the various ranges of the distance between the lateral edge of the flange and the hinge point and avoid careless osteotomy of this area in biplanar OWHTO.

## Conclusions

In biplanar OWHTO, anterolateral cortex osteotomy would be required. However, the range of the required anterolateral cortex osteotomy distance varied widely and the required anterolateral cortex osteotomy distance depended on the flange thickness and the angle between the transverse and ascending cuts. In addition, change of the ascending cut plane can change the necessity of anterolateral cortex osteotomy.

## Data Availability

The datasets used and/or analysed during the current study available from the corresponding author on reasonable request.

## Abbreviations

OWHTO: Open wedge high tibial osteotomy
OA: Osteoarthritis
CT: Computed tomography
ICCs: Intraclass correlation coefficients
PSI: Patient specific instrumentation
